# ETS1–EGR2 facilitates renal cell carcinoma progression by activating NOTCH signalling

**DOI:** 10.1002/ctm2.70778

**Published:** 2026-08-17

**Authors:** Weifeng Ye, Sishun Gan, Qiang Xu, Xiaoyang Shu, Panpan Dong, Lin Li, Chao Tang

**Affiliations:** ^1^ Department of Pharmacy Children's Hospital, Zhejiang University School of Medicine, National Clinical Research Center for Children and Adolescents' Health and Disease Hangzhou China; ^2^ Children's Hospital Zhejiang University School of Medicine, National Clinical Research Center for Children and Adolescents' Health and Disease Hangzhou China; ^3^ Department of Urology Xinhua Hospital, Shanghai Jiaotong University, School of Medicine Shanghai China; ^4^ Department of Oncology Jingzhou Hospital Affiliated to Yangtze University Jingzhou China; ^5^ School of Basic Medical Sciences, Health Science Center, Yangtze University Jingzhou China; ^6^ Department of Reproductive Immunology Tongde Hospital of Zhejiang Province Hangzhou China; ^7^ Department of Urology The Fourth Affiliated Hospital of Soochow University, Suzhou Dushu Lake Hospital Medical Center of Soochow University Suzhou China; ^8^ Department of Urology Third Affiliated Hospital of Naval Medical University Shanghai China

**Keywords:** ccRCC, ETS1, EGR2, NOTCH, target therapy

## Abstract

**Background:**

Renal cell carcinoma (RCC) is known as one of the three major malignant tumors of the urinary system, and clear‐cell RCC (ccRCC) is the most frequent and aggressive pathological subtype and is correlated with high mortality. Despite diverse treatments, the clinical prognosis of patients with RCC remains poor. Therefore, further characterization of the mechanisms underlying ccRCC progression is urgently required.

**Methods:**

Different RCC celllines were used to study cell malignancies in vitro and to elucidate mechanisms. Nude mice were used for validating results in vivo. Sequencing, database and bioinformatic analysis were applied for screening candidate genes and signalings. Clinical samples were used to verify the expression pattern of key genes.

**Results:**

ETS1 expression is significantly higher in clinical ccRCC tissue samples, and consistent withthese findings, ETS1 overexpression increases RCC cell malignancies in cultured cell models in vitro as well as tumor growth in nude mice in vivo. Mechanistically, ETS1 binds to EGR2 and destabilizes the EGR2 protein, and the decreased EGR2 levels mitigate its inhibitory effect on NOTCH signaling through *RBP‐J*, resulting in activation of the NOTCH signaling pathway, ultimately leading to RCC development.

**Conclusion:**

Therefore, targeting the ETS1‒EGR2‒NOTCH signaling axis may be a promising therapeutic strategy for ccRCC.

**Key points:**

- ETS1 expression is significantly higher in ccRCC samples, and ETS1‐overexpression increases RCC cell malignancies in vitro and tumour growth in nude mice.
- ETS1 binds to EGR2 and destabilises EGR2 protein, which relieves EGR2's inhibitory effect on NOTCH signalling.
- NOTCH signalling activation promotes RCC development.
- The ETS1‒EGR2‒NOTCH axis would potentially be a promising therapeutic strategy in ccRCC.

## Graphical abstract

1. ETS1 expression is significantly higher in ccRCC samples, and ETS1‐overexpression increases RCC cell malignancies in vitro and tumour growth in nude mice.
2. ETS1 binds to EGR2 and destabilises EGR2 protein, which relieves EGR2's inhibitory effect on NOTCH signalling.
3. NOTCH signalling activation promotes RCC development.
4. The ETS1‒EGR2‒NOTCH axis would potentially be a promising therapeutic strategy in ccRCC.

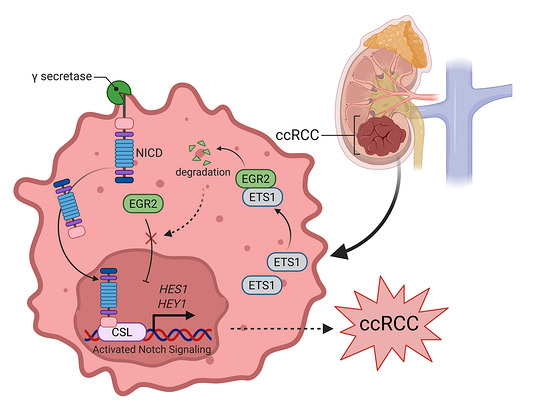

## 1 INTRODUCTION

The global incidence of renal cell carcinoma (RCC) has been rising persistently over the past decades. This malignancy makes up 3%‒5% of all adult malignant tumours and stands as the sixth primary contributor to cancer‐associated mortality worldwide. Among all RCC types, clear‐cell RCC (ccRCC) is recognised as the most aggressive pathological subtype.^1^, ^2^ Owing to the asymptomatic nature of RCC, diagnosis of the disease often happens only after metastases has already begun. Moreover, distant metastasis is a significant obstacle in the effective treatment of ccRCC.^3^

Although diverse treatments are applied in clinical practice, such as radiotherapy, chemotherapy and targeted therapies to control ccRCC development, patient clinical prognosis remains unfavourable due to therapeutic resistance and severe adverse effects induced by drug treatment.^4^, ^5^ Despite advances in novel strategies based on immune checkpoint inhibitors, such as nivolumab,^6^ there is still no tumour regression or cure, and the long‐term prognosis remains unsatisfactory. Therefore, there is an urgent need to further characterise the mechanism of ccRCC progression and develop novel and effective therapeutic strategies.

As a member of the extensive ETS‐domain transcription factor superfamily, erythroblast transformation‐specific 1 (ETS1) exerts vital regulatory functions throughout the initiation and progression of diverse human malignancies.^7^, ^8^ Possessing a unique DNA‐binding structural domain, ETS1 predominantly functions as a transcriptional activator. This nuclear protein has a molecular mass of 54 kDa. Recent studies have also revealed its effect on repressing transcription of genes such as *Il10*.^9^ The association of ETS1 with different carcinomas and its regulatory role have been explored, showing that ETS1 is strongly expressed in various cancers such as hepatocellular carcinoma,^10^ gastric cancer,^11^ ovarian cancer^12^ and breast cancer.^13^ Silencing of ETS1 expression could effectively restrain cell proliferation, suppress invasion and migration but induce cell apoptosis in these cancers, indicating characterisation of ETS1 as an oncogenic factor and its potential as a biomarker for carcinomas.

As a zinc‐finger transcription factor responsive to mitogenic, hypoxic and inflammatory stimuli, early growth response 2 (EGR2) exhibits context‐dependent dual functions as either a tumour suppressor or oncogenic driver across diverse malignancies during cancer progression,^14^ with its divergent bioactivities largely dictated by tumour type, intracellular signalling stimuli and epigenetic regulation. Moreover, EGR2 participates in reciprocal crosstalk with key oncogenic pathways such as the phosphatase and tensin homolog (PTEN) signalling,^15^, ^16^ further amplifying its regulatory influence on tumour stemness, immune evasion and stromal‒tumour crosstalk.

Accumulated clinical transcriptomic data, patient specimen analyses and functional preclinical studies have firmly established multifaceted oncogenic roles of dysregulated NOTCH signalling in driving RCC, predominantly ccRCC progression at every major disease stage.^17^, ^18^ Constitutive hyperactivation of core NOTCH cascade components, including NOTCH receptors, Jagged/DLL ligands and nuclear NOTCH1 intracellular domain (NICD) fragments, is widely detected in primary RCC tumours relative to matched normal renal tissues, with elevated pathway activity correlating with higher pathological grade, advanced ‌tumour‒node‒metastasis (TNM) staging and increased metastatic risk.

Here, we report that ETS1 expression is significantly higher in ccRCC tissues than in adjacent normal kidney tissues, and that, consistent with these findings, upregulated ETS1 expression aggravates the malignant phenotypes of RCC, which encompasses in vitro proliferative capacity, migratory and invasive abilities, and clonogenic potential, alongside in vivo tumour outgrowth in nude mouse xenograft models. Mechanistically, ETS1 binds to EGR2, thereby destabilising the EGR2 protein, which in turn diminishes the inhibitory effect of EGR2 on NOTCH signalling, resulting in the activation of the NOTCH signalling pathway and ultimately leading to RCC development. Therefore, the ETS1‒EGR2‒NOTCH signalling axis may be a promising therapeutic target for ccRCC.

## 2 MATERIALS AND METHODS

### 2.1 Cell line and cell culture

Two cell lines, ACHN and 786O, were sourced from the Chinese Academy of Sciences. For cultivation, 786O cells were cultured in Hyclone RPMI‐1640 medium containing an identical concentration of 10% (v/v) foetal bovine serum (FBS, Life Technologies), and ACHN cells were maintained in Hyclone‐supplied MEM medium mixed with 10% FBS. Consistent with prior protocols,^19^ both cell lines were grown into monolayers on plastic culture plates within a humidified incubator set to 37°C and 5% CO_2_.

### 2.2 Antibodies and chemicals

ETS1 (AF6812), HES1 (AF2167), Flag (AF0036) and GST (AF0174) antibodies were from Beyotime Biotechnology. HEY1 antibody (bs‐16500R) and RBP‐J antibody (bs‐2966R) were obtained from Bioss Biotechnology, and GAPDH (sc‐32233) and αTubulin (sc‐32293) antibodies were acquired from Santa Cruz Biotechnology. DAPT (S2215), cycloheximide (S7418) and MG132 (S2619) were acquired from Selleckchem.

### 2.3 Dual‐luciferase reporter assays

NOTCH‐specific luciferase reporter activity was detected via dual‐luciferase reporter assays. All experimental operations were performed with the commercial kit purchased from Promega, in accordance with previously established experimental protocols.^20^ The Renilla luciferase plasmid served as an internal control to normalise firefly luciferase signals. All quantitative data were derived from three separate biological replicates, with each experiment conducted in technical triplicate. Empty plasmids, constructs encoding scrambled shRNA and vehicle solution were separately included as controls.

### 2.4 Total RNA extraction and quantitative real‐time PCR

Total RNA was extracted from 786O cells, ACHN cells and tissue specimens using TRIzol reagent (Takara Biotechnology) as per the manufacturer's instructions, consistent with a prior study.^21^ For first‐strand cDNA synthesis, 5 µg of total RNA diluted in a 20 µL reaction system was reverse‐transcribed using SuperScript III and oligo(dT) primers at 42°C for 60 min. Quantitative real‐time PCR was subsequently performed on the synthesised cDNA to quantify the transcript levels of target genes, including *ETS1*, *EGR2*, *RBP‐J*, *HES1* and *HEY1*. *GAPDH* gene was employed as an internal reference gene to normalise the mRNA abundance of each target gene. Relative mRNA expression fold alterations were quantified using the 2^− ΔΔCt^ calculation method, consistent with previously published methodologies.^22^

### 2.5 CRISPR/Cas9 construction

SgRNA expression plasmids targeting human *ETS1* were constructed based on the pX330 backbone. Candidate target sequences were designed via the *CRISPR* DESIGN platform. All validated targeting fragments were amplified, inserted into vectors, and confirmed by Sanger DNA sequencing. The pX330‐sg*ETS1* construct was co‐transfected with a puromycin resistance plasmid into 786O cells using Lipofectamine transfection reagent (Invitrogen). Transfected cells were subjected to 7 days of puromycin selection at a concentration of 2 µg/mL (Invitrogen), followed by continuous expansion in standard complete culture medium.

### 2.6 Protein extraction, Western blot and co‐immunoprecipitation

Protein was extracted from 786O cells, ACHN cells, human clinical RCC specimens and mouse xenograft tissues using commercial RIPA lysis buffer from Beyotime Biotechnology. After protein quantification, Western blot analysis was carried out in accordance with a previously published protocol.^23^ In short, an equal amount of 50 µg total protein from each specimen was resolved through sodium dodecyl sulfate‐polyacrylamide gel electrophoresis (SDS‒PAGE) electrophoresis, followed by electrophoretic transfer to polyvinylidene fluoride (PVDF) membranes supplied by Millipore. After membrane blocking, primary antibodies targeting proteins of interest were applied for incubation, followed by matching secondary antibodies purchased from Beyotime Biotechnology. Target protein bands were detected with an enhanced chemiluminescence imaging system. Band gray values were quantified with ImageJ software. GAPDH or αTubulin was used as internal reference signal.

Co‐immunoprecipitation (Co‐IP) experiments were performed following an established protocol.^24^ In short, harvested 786O cells were lysed with immunoprecipitation (IP)‐specific lysis buffer, and the lysates were incubated with designated antibodies together with protein A/G plus agarose beads. The agarose beads were rinsed five times with IP lysis buffer, and captured immune complexes were eluted using Western blot loading buffer. Whole‐cell lysates and IP products were subsequently analysed by Western blot using the identical procedure mentioned above.

### 2.7 GST pull‐down assays

Briefly, cell lysates overexpressing Flag‐tagged EGR2 were mixed with *Escherichia coli*‐purified recombinant GST or GST‒ETS1 fusion proteins, followed by incubation in IP buffer at 4°C for 30 min. Glutathione sepharose beads were then added to capture GST fusion proteins via pull‐down assays. After four rounds of washing with phosphate‐buffered saline (PBS) containing .2% Tween‐20, the beads were resuspended in SDS protein loading buffer to elute bound proteins. Eluted protein samples were subject to Western blot using an anti‐Flag antibody.

### 2.8 Cell‐counting kit‐8 assays

Cell‐counting kit‐8 (CCK‐8) assays were carried out to detect cell viabilities as detailed previously.^25^ In brief, cells (in 96‐well plates) were transfected with ETS1‐expressing, NICD‐expressing and EGR2‐expressing plasmids, or ETS1 shRNA, EGR2 shRNA or control vectors in combination with or without DAPT treatment. After culture for 48 or 72 h, 20 µL CCK‐8 reagent was added into each well to co‐culture for another 2 h. Subsequently, absorbance values at 450 nm were recorded with a multi‐functional microplate reader (Bio‐Tek Instruments) to evaluate cellular viability.

### 2.9 Wound healing assays

Wound healing assays were conducted as previously described.^26^ In short, 4 × 10^5^ ACHN or 786O cells were seeded in six‐well plates and were transfected with ETS1‐expressing plasmid, NICD‐expressing, EGR2‐expressing, ETS1 shRNA, EGR2 shRNA or control vectors in combination with or without DAPT treatment. Twenty‐four hours after transfection, cells were subject to serum starvation for 12 h. After rinsing with medium to remove unattached cells, sterile pipette tips were applied to introduce uniform artificial scratches on the cell monolayer. After 24 h of incubation, cell migration into the scratch wound area was visualised under a microscope, and the width of the wound gap at each field was quantified with ImageJ software.

### 2.10 Matrigel invasion assays

The invasive potential of 786O and ACHN cells was evaluated using Matrigel‐precoated Transwell chambers (Costar, Cambridge) with 8 µm‐pore polycarbonate membranes and a 6.5 mm insert diameter (BD Biosciences), following a previously established protocol.^19^ Briefly, each Transwell membrane was coated with 50 µL of Matrigel diluted in complete medium at a 1:2 volume ratio. Forty‐eight hours post‐transfection, 2 × 10^5^ suspended cells in 200 µL medium (without serum) were added into the upper compartment of each insert. The lower chamber was filled with 600 µL medium supplemented with 10% FBS to act as a chemoattractant. After a further 24 h culture, non‐invasive cells retained on the apical membrane surface were carefully wiped away. Invaded cells adhering to the basolateral side of the filter were fixed and then stained with .2% crystal violet solution. For quantification, invasive cell counts were acquired from three random microscopic fields per chamber, with three biological replicate chambers analysed per experimental group; cell enumeration was performed under an optical light microscope.

### 2.11 Colony formation assays

Colony formation assays were conducted as per a previously published protocol.^27^ Briefly, 786O and ACHN cells were transduced with the designated lentiviruses encoding overexpression constructs and/or shRNA sequences. 24 h post‐transduction, cells were seeded into six‐well plates (1000 cells each well) with 2 mL culture medium. Cells were cultured for 2 weeks in the presence or absence of DAPT treatment. Subsequent fixation of cell colonies was performed with methanol at ambient temperature over a 10‐min incubation, prior to staining treatment with .5% crystal violet solution. Finally, stained colonies were imaged and counted, and quantitative colony counts were subjected to statistical analysis.

### 2.12 Chromatin immunoprecipitation‐PCR assays

Chromatin immunoprecipitation‐PCR (ChIP‐PCR) experiments were performed using a commercially available ChIP kit (Millipore) according to a previously described protocol.^24^ In short, chromatin DNA was fragmented via sonication to yield 300‒400 bp DNA fragments, which were subsequently subjected to IP using an anti‐EGR2 antibody or control immunoglobulin G (IgG). The immunoprecipitated mixtures were co‐incubated with protein A agarose beads pre‐bound to salmon sperm DNA. The antibody‒chromatin‒agarose precipitates underwent gentle rinsing and collection, after which reverse cross‐linking reactions were carried out. Genomic DNA fragments released upon cross‐link reversal were isolated via a commercially available DNA purification kit and subjected to downstream PCR amplification.

### 2.13 Immunohistochemistry staining

Immunohistochemistry (IHC) experiments were carried out in accordance with previously published protocols.^19^, ^28^ Briefly, freshly collected human ccRCC tissues and paired adjacent normal renal tissues were washed with pre‐cooled PBS, followed by overnight fixation in 4% paraformaldehyde at 4°C. The next day, fixed tissue specimens underwent dehydration, paraffin embedding and serial sectioning (4 µm). After deparaffinisation and rehydration, endogenous peroxidase activity was inhibited by incubation with methanolic .3% hydrogen peroxide. Antigen retrieval was achieved by heating the slides in pH 6.0 citrate buffer via boiling for 15 min. Next, sections were subject to incubation overnight at 4°C with corresponding primary antibodies (ETS1 or EGR2), while normal IgG served as the negative control. Immunoreactive signals were visualised using a commercial detection kit purchased from Zsbio.

Quantification of ETS1 and EGR2 immunoreactivity was carried out by evaluating three non‐overlapping microscopic fields per specimen. The staining scores were assigned according to the proportion and intensity of positively stained cells as follows: 0, no positive cells; 1, weak staining in <50% of cells; 2, strong staining in <50% of cells; 3, weak staining in ≥50% of cells; 4, strong staining in ≥50% of cells.

### 2.14 Xenografts assay

Six‐week‐old female BALB/c nude mice were housed under specific pathogen‐free conditions with 50%‒60% humidity and a 12 h light/12 h dark diurnal cycle. Animals were randomly allocated to distinct experimental groups, with six mice per group. To establish subcutaneous xenograft tumour models, 5 × 10^6^ 786O cells transduced with control lentivirus or lentiviruses overexpressing ETS1, EGR2 or NICD were suspended in 100 µL PBS and subcutaneously injected into the left axilla of each mouse, following a previously described protocol,^29^ and the mice were administrated with or without DAPT intraperitoneally. Once any individual tumour volume reached 1000 mm^3^ post‐inoculation, all mice were euthanised. Subcutaneous xenograft tumours were dissected and harvested, and their volume (*V*) and weight were quantified by researchers blinded to group assignments. All animal housing, care and experimental manipulations were reviewed and approved by the Institutional Animal Care and Use Committee of Zhejiang University, and all operations strictly complied with the ARRIVE 2.0 reporting guidelines.

### 2.15 RNA‐sequencing analysis

Total RNA (≥3 µg) extracted from ETS1‐overexpressing 786O cells was subjected to oligo(dT) enrichment. Purified mRNA was fragmented with fragmentation buffer, and double‐stranded cDNA was reverse‐transcribed using the fragmented mRNA templates. The resultant cDNA underwent end repair and ligation with Illumina sequencing adapters, followed by agarose gel size selection to recover fragments of approximately 250 bp and subsequent PCR amplification. The constructed cDNA library was sequenced on the Illumina HiSeq 2000 platform. Raw sequencing reads were mapped to transcript sequences, and transcript expression abundances were quantified as reads per kilobase of exon per million mapped reads (RPKM). Only uniquely mapped exon reads were included for RPKM calculation. Differential expression analysis was performed via Cufflinks. Genes with log_2_(fold change) >1 or <−1 and *p* < .05 between the control and ETS1‐overexpression groups were defined as significantly differentially expressed genes. Raw data of sequencing can be made available on reasonable request.

On the other hand, the widely used annotated gene database gene ontology (GO) and kyoto encyclopedia of genes and genomes (KEGG) were used to perform pathway enrichment analysis on the differential genes using the CusterProfiler software. For KEGG analysis, pathway enrichment results were considered significantly enriched when the *p*‐value was less than .05. For GO analysis, pathway enrichment results were considered significantly enriched when the *p*‐value was less than .05.

### 2.16 LC‒MS/MS analysis

Peptide pellets were resuspended with mobile phase A consisting of .1% formic acid, followed by chromatographic separation on the EASY‐nLC 1200 instrument manufactured by Thermo Scientific. The nanoflow LC separation ran at a steady flow speed of 400 nL/min using a linear gradient program of mobile phase B (.1% formic acid dissolved in 80% acetonitrile): the proportion rose from 2% to 7% within 1 min, increased to 35% over the subsequent 35 min, shifted to 55% in another 9 min, raised to 100% in 7 min, and sustained at 100% for the last 8 min of the separation run.

Peptides eluted from the liquid chromatography column were ionised through a nano‐electrospray ion source and subjected to detection with a Q Exactive HF‐X mass spectrometer, with electrospray voltage fixed at 2.0 kV. Intact peptide precursor ions were captured via full mass spectrometry (MS) scanning in the Orbitrap detector at a resolving power of 60 000. Precursor molecules were fragmented through HCD with NCE adjusted to 27, and the resultant fragment ions were measured in the same Orbitrap analyser with 15 000 resolution. Mass spectrum acquisition adopted the DDA strategy: the 20 precursor ions showing the highest signal intensity in each full scan were successively chosen for secondary MS detection, and a 20‐s dynamic exclusion window was configured to avoid repeated ion selection.

The raw MS files were imported into Proteome Discoverer 2.4 (Thermo Scientific) for data processing and database matching against the human Swiss‐Prot database (version 2023_09). Trypsin/P was chosen as the digestive enzyme, allowing up to two incomplete cleavage sites per peptide sequence. The mass tolerance cutoffs were configured as 10 ppm for parent ions and .02 Da for fragment ion species. Cysteine carbamidomethylation was designated a fixed chemical alteration, whereas methionine oxidation and protein N‐terminal acetylation were classified as adjustable variable modifications. Only peptides assigned high confidence scores were screened out for subsequent quantitative and functional analysis.

### 2.17 Bioinformatics analysis

The GEPIA database at http://gepia.cancer‐pku.cn was utilised to compare ETS1 expression [log_2_(TPM + 1)] between tumour renal clear‐cell carcinoma (KIRC, T, *n* = 523) and matched normal kidney tissues (KIRC, N, *n* = 100) based on the KIRC dataset. Meanwhile, Kaplan‒Meier disease‐free survival (DFS) curves were generated with the median expression value as the grouping cutoff (top 50% high expression vs. bottom 50% low expression), and 95% confidence intervals were calculated for survival comparisons. The correlations between *ETS1* and *HES1* expression, *ETS1* and *HEY1* expression, *EGR2* and *HES1* expression, and *EGR2* and *HEY1* expression in RCC patient were assessed and analysed using auto‐best cut‐off.

Transcriptomic expression profiles of RCC cell lines were retrieved from the Cancer Cell Line Encyclopaedia (CCLE) database, which is accessible at https://depmap.org/portal/data_page/?tab=allData. All statistical calculations were performed with R (v4.0.3). Any outcome with a *p*‐value below .05 was defined as statistically significant.

For ETS1‐high and ETS1‐low analysis, we obtained a total of 208 columns of gene expression data from ccRCC tissues and 208 columns of data from normal tissues from the GEO database. GSE40435 (based on GPL10558) contains 101 columns of ccRCC tissue data and 101 columns of normal tissue data; GSE53757 (based on GPL570) contains 72 columns of ccRCC tissue data and 72 columns of normal tissue data; GSE66272 (based on GPL570) contains 26 columns of ccRCC tissue data and 27 columns of normal tissue data; GSE781 (based on GPL97) contains nine columns of ccRCC tissue data and eight columns of normal tissue data. All the aforementioned data were corrected using the limma package, followed by merging and batch correction of all data using the sva package.

### 2.18 GSEA enrichment analysis and diagnostic model construction

We downloaded the signature gene sets from the MSigDB database (https://www.gsea‐msigdb.org/gsea/msigdb). First, the batch‐processed transcriptome data obtained from the GEO database was divided into ETS1 high‐expression group and ETS1 low‐expression group for gene set enrichment analysis (GSEA) enrichment analysis to evaluate the gene molecular functions and pathways related to ccRCC survival, and the top five most significant ones were selected for display. Subsequently, clinical data from TCGA patients were used to draw diagnostic ROC curves, and the calibration method in the pROC package was applied for calculation. All statistical analyses were carried out using R software (version 4.4.1), with the significance level defined as *p* < .05.

### 2.19 Obtainment of tissue samples

Ten matched tissue pairs containing ccRCC lesions and corresponding peritumoural normal tissues were harvested from surgical patients treated at the Third Affiliated Hospital of Naval Medical University during 2024‒2025. All recruited subjects did not receive any chemotherapy or radiation therapy before surgical excision. Experienced pathologists performed histological identification to confirm the pathological characteristics of all tumour and paired surrounding normal tissues. The whole experimental scheme was examined and authorised by the Institutional Review Board of the Third Affiliated Hospital of Naval Medical University, and all participants provided signed informed consent prior to tissue sampling.

### 2.20 Statistical analysis

All the data were expressed as mean ± SD and were analysed by unpaired Student's *t*‐test between two groups or with one‐way ANOVA and Tukey‒Kramer multiple comparison test (SPSS 13.0 J software; SPSS, Inc.) among multiple groups. Statistical significance was assessed at *p* < .05 and <.01.

## 3 RESULTS

### 3.1 ETS1 is highly expressed in ccRCC tissues and promotes RCC cell malignancies

**FIGURE 1:**
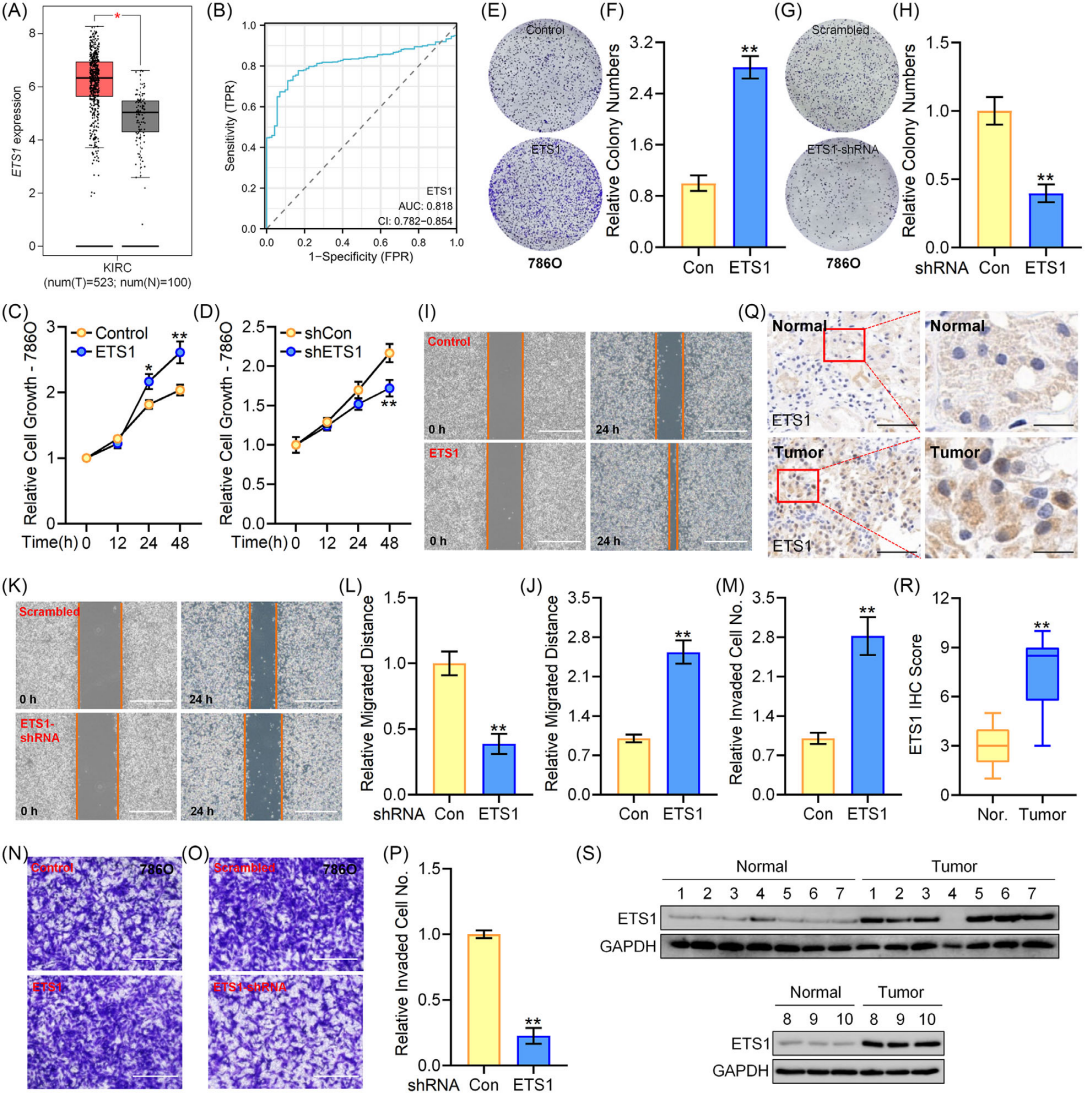
Erythroblast transformation‐specific 1 (ETS1) is highly expressed in clear‐cell renal cell carcinoma (ccRCC) tissues and promotes RCC cell malignancies. (A) Expression of *ETS1* in KIRC tissues (T) and control groups (N) was revealed by GEPIA database. (B) ROC panel showing EST1 expression with an AUC = .818. (C) Cell‐counting kit‐8 (CCK‐8) assays of 786O cells transfected with an ETS1‐expressing vector (ETS1) or a control empty vector (control) and cultured for the indicated time periods. (D) CCK‐8 assays of 786O cells transfected with an ETS1 shRNA‐expressing vector (shETS1) or a control empty vector (shCon) and cultured for the indicated time periods. (E) Colony formation assays of 786O cells infected with lentiviruses expressing ETS1 or with control lentiviruses (control). (F) Quantitative analysis of relative colony numbers in (E). (G) Colony formation assays of 786O cells infected with lentiviruses expressing ETS1 shRNA or with control lentiviruses (scrambled). (H) Quantitative analysis of relative colony numbers in (G). (I) Wound healing assays of 786O cells transfected with an ETS1‐expressing vector (ETS1) or a control empty vector (control) at 24 h. Bar, 100 µm. (J) Statistical analysis of migrated distance in (I). (K) Wound healing assays of 786O cells transfected with an ETS1 shRNA‐expressing vector (ETS1 shRNA) or a control empty vector (scrambled) at 24 h. Bar, 100 µm. (L) Statistical analysis of migrated distance in (K). (M) Statistical analysis of invaded cell number in (N). (N) Matrigel invasion assays of 786O cells transfected with an ETS1‐expressing vector (ETS1) or a control empty vector (Control) at 24 h. Bar, 100 µm. (O) Matrigel invasion assays of 786O cells transfected with an ETS1 shRNA‐expressing vector (ETS1 shRNA) or a control empty vector (scrambled) at 24 h. Bar, 100 µm. (P) Statistical analysis of invaded cell number in (O). (Q) Immunohistochemistry (IHC) staining for ETS1 by using paraffin‐embedded sections of cancer tissues and adjacent normal epithelial tissues from patients with ccRCC. *N*  =  10; bar, 20 µm. (R) IHC score for ETS1 in (Q). *N*  =  10. (S) The protein expression of ETS1 in cancer tissues (tumour) and adjacent normal epithelial tissues (normal) from patients with ccRCC. ^*^ *p* < .05; ^**^ *p* < .01; error bar, SD.

First, to explore the potential role of ETS1 in RCC, we examined the expression profile of ETS1 in KIRC using the GEPIA database. *ETS1* expression was found to be significantly elevated in KIRC tissues (T) compared to that in normal tissues (N) (Figure 1A). Consistently, the ROC panel showed EST1 expression with an AUC of .818 (range: .8‒.9), suggesting that ETS1 expression may have a high diagnostic value (Figure 1B). Similarly, tumours with high ETS1 expression were associated with blood vessel morphogenesis, tube development and vasculature development, suggesting a higher metastatic potential (Figure S1A,B). To determine whether ETS1 regulates RCC progression, we used 786O RCC cell line, and upregulated or downregulated ETS1 expression by transfection with an ETS1‐expressing plasmid or ETS1 shRNA, respectively. As expected, ETS1 overexpression significantly induced cell proliferation, particularly at 24 and 48 h (Figure 1C), and promoted colony formation (Figure 1E,F), whereas silencing of ETS1 using ETS1 shRNA effectively suppressed both ETS1 mRNA and protein expression (Figure S1C,D), significantly decreasing cell proliferation and colony formation at 48 h (Figure 1D,G,H). Consistently, the ectopic expression of ETS1 also significantly potentiated the migratory and invasive potential of RCC cells (Figure 1I,J,M,N), and conversely, suppression of ETS1 expression exerted the opposite effects (Figure 1K,L,O,P), demonstrating that ETS1 expression affects the biological behaviour of RCC cells. Furthermore, the impact of ETS1 knockdown on RCC cells was validated in *ETS1*‐knockout 786O cells, showing that *ETS1* deficiency significantly hampered cell proliferation, migration, invasion and colony formation (Figure S1F‒L). In addition, the clinical implications of ETS1 expression were further verified by comparing ETS1 protein expression in 10 paired ccRCC and non‐tumour tissues. Both the staining data and Western blotting results revealed that ETS1 protein expression was significantly higher in the tumour samples than in the normal control group (Figure 1Q‒S), which was consistent with the mRNA expression pattern of *ETS1* in the KIRC database (Figure 1A). These data indicate that aberrantly upregulated ETS1 expression is correlated with ccRCC progression, where ETS1 plays a tumour‐stimulating role.

### 3.2 ETS1 regulates biological malignancies in RCC cells through Notch signalling

**FIGURE 2:**
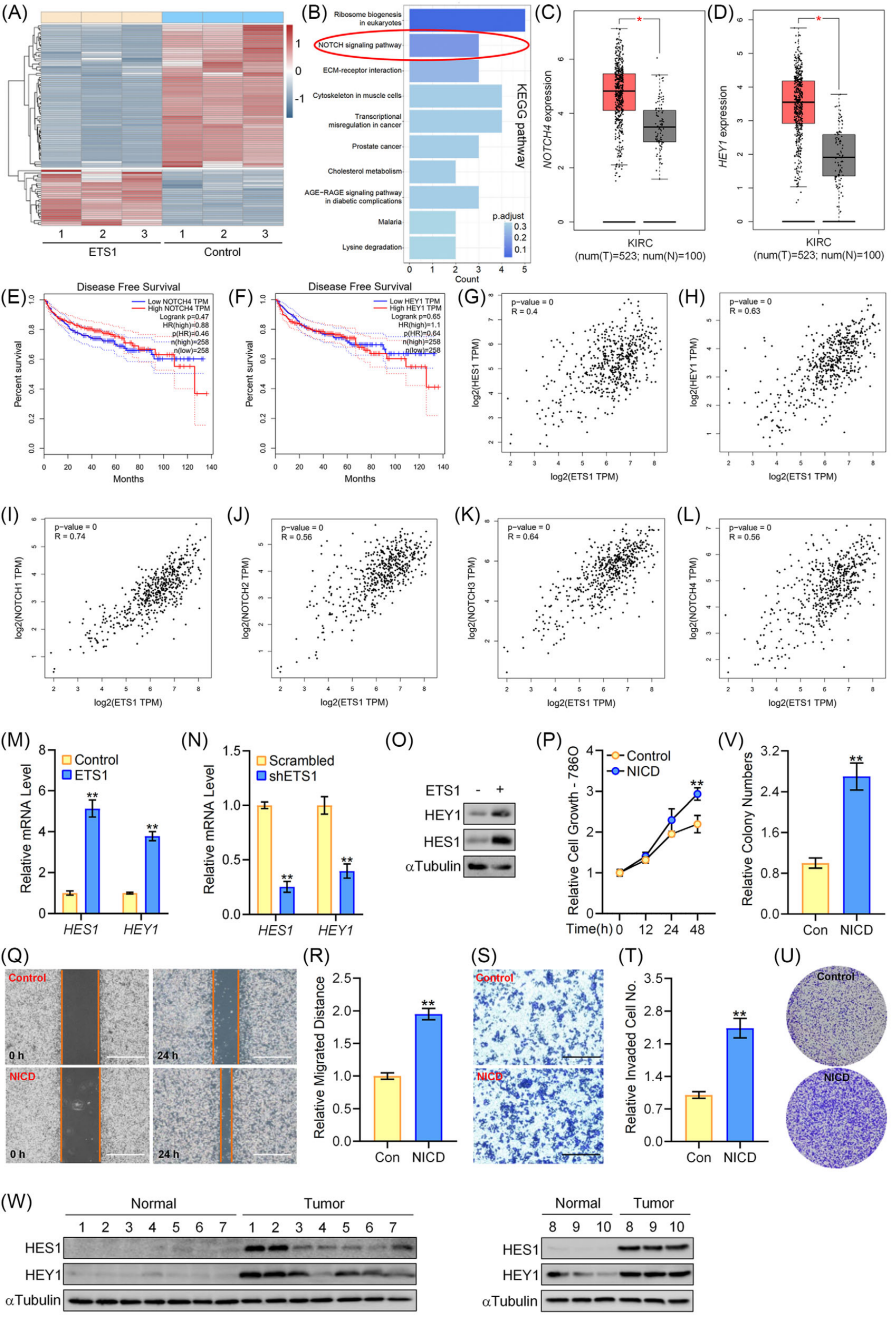
Erythroblast transformation‐specific 1 (ETS1) through Notch signalling regulates renal cell carcinoma (RCC) cell biological malignancies. (A) 786O cells were transfected with an ETS1‐expressing vector (ETS1) or a control empty vector (Con) and were subject to RNA‐sequencing. A heatmap for the altered genes was shown. (B) KEGG analysis was conducted to compare the gene expression from ETS1‐overexpression group and the control. (C) Expression of *NOTCH4* in KIRC tissues (T) and control groups (N) was revealed by GEPIA database. (D) Expression of *HEY1* in KIRC tissues (T) and control groups (N) was revealed by GEPIA database. (E) Kaplan‒Meier analysis of disease free survival for KIRC patients with altered expression of *NOTCH4*. (F) Kaplan‒Meier analysis of disease free survival for KIRC patients with altered expression of *HEY1*. (G) GEPIA database displayed the correlation between *ETS1* and *HES1*. (H) GEPIA database displayed the correlation between *ETS1* and *HEY1*. (I) GEPIA database displayed the correlation between *ETS1* and *NOTCH1*. (J) GEPIA database displayed the correlation between *ETS1* and *NOTCH2*. (K) GEPIA database displayed the correlation between *ETS1* and *NOTCH3*. (L) GEPIA database displayed the correlation between *ETS1* and *NOTCH4*. (M) The mRNA levels of *HES1* and *HEY1* in 786O cells transfected with an ETS1‐expressing vector (ETS1) or a control empty vector (control) for 24 h. (N) The mRNA levels of *HES1* and *HEY1* in 786O cells transfected with an ETS1 shRNA‐expressing vector (shETS1) or a control empty vector (scrambled) for 48 h. (O) The protein levels of HES1 and HEY1 in 786O cells transfected with an ETS1‐expressing vector (ETS1, +) or a control empty vector (ETS1, ‒) for 24 h. (P) Cell‐counting kit‐8 (CCK‐8) assays of 786O cells transfected with a NOTCH1 intracellular domain (NICD)‐expressing vector (NICD) or a control empty vector (control) and cultured for the indicated time periods. (Q) Wound healing assays of 786O cells transfected with a NICD‐expressing vector (NICD) or a control empty vector (control) at 24 h. Bar, 100 µm. (R) Statistical analysis of migrated distance in (Q). (S) Matrigel invasion assays of 786O cells transfected with a NICD‐expressing vector (NICD) or a control empty vector (control) at 24 h. Bar, 100 µm. (T) Statistical analysis of invaded cell number in (S). (U) Colony formation assays of 786O cells infected with lentiviruses expressing NICD or with control lentiviruses (control). (V) Quantitative analysis of relative colony numbers in (U). (W) The protein expression of HES1 and HEY1 in cancer tissues (tumour) and adjacent normal epithelial tissues (normal) from patients with clear‐cell renal cell carcinoma (ccRCC). ^*^ *p* < .05; ^**^ *p* < .01; error bar, SD.

To dissect the molecular mechanisms through which ETS1 exerts its biological functions in ccRCC, we performed RNA‐sequencing (RNA‐seq) on 786O cells stably transfected with ETS1 overexpression plasmid or empty control vector (Con). In total, 96 transcripts displayed significantly altered expression following ETS1 overexpression, defined by thresholds of log_2_(fold change) > 1 or <−1. Among these differentially expressed transcripts, 27 were upregulated and 69 were downregulated (Figures 2A and S2A), suggesting that variations in ETS1 expression markedly altered the transcriptome. KEGG analysis revealed that ETS1 overexpression affected many pathways, including the Notch signalling pathway (Figure 2B). Consistently, as indicated by the GEPIA database, the expression of Notch ligands, such as *NOTCH4* and its target *HEY1*, was significantly increased in KIRC tissues (T) than in normal tissues (N) (Figure 2C,D). Notably, patients with KIRC with higher expression of either *NOTCH4* or *HEY1* exhibited a lower DFS, although the difference was not statistically significant (Figure 2E,F). Moreover, a positive correlation between the expression of *ETS1* and all four NOTCH receptors (*NOTCH1*, *NOTCH2*, *NOTCH3* and *NOTCH4*) as well as targets *HES1* and *HEY1* was found in the GEPIA database (Figure 2G‒L), thereby providing further evidence that ETS1 contributes to NOTCH signalling activation. Moreover, by further stratifying patients into ETS1‐high and ETS1‐low groups, the NOTCH signalling pathway was found to be enriched in the ETS1‐high tumour group (Figure S2B,C). Indeed, ETS1 overexpression not only significantly induced RBP‐Jk‐Luciferase (Luc) reporter activity but also enhanced the mRNA and protein expression of the targets HES1 and HEY1 (Figures 2M,O and S2C), whereas ETS1 knockdown significantly decreased RBP‐Jk‐Luc reporter activity and suppressed both *HES1* and *HEY1* expression in RCC cells (Figures 2N and S2D). As expected, activation of NOTCH signalling by overexpression of the human NICD significantly promoted cell proliferation, mobility (migration and invasion) and colony number (Figure 2P‒V), which was consistent with a previous report showing that activation of NOTCH signalling causes RCC development.^30^ Consistently, Western blotting revealed that HES1 and HEY1 protein expression was significantly higher in tumour samples than in the normal control group (Figure 2W). Thus, these results indicate that ETS1 contributes to ccRCC progression likely through the activation of NOTCH signalling.

### 3.3 ETS1 binds to EGR2 and destabilises EGR2

**FIGURE 3:**
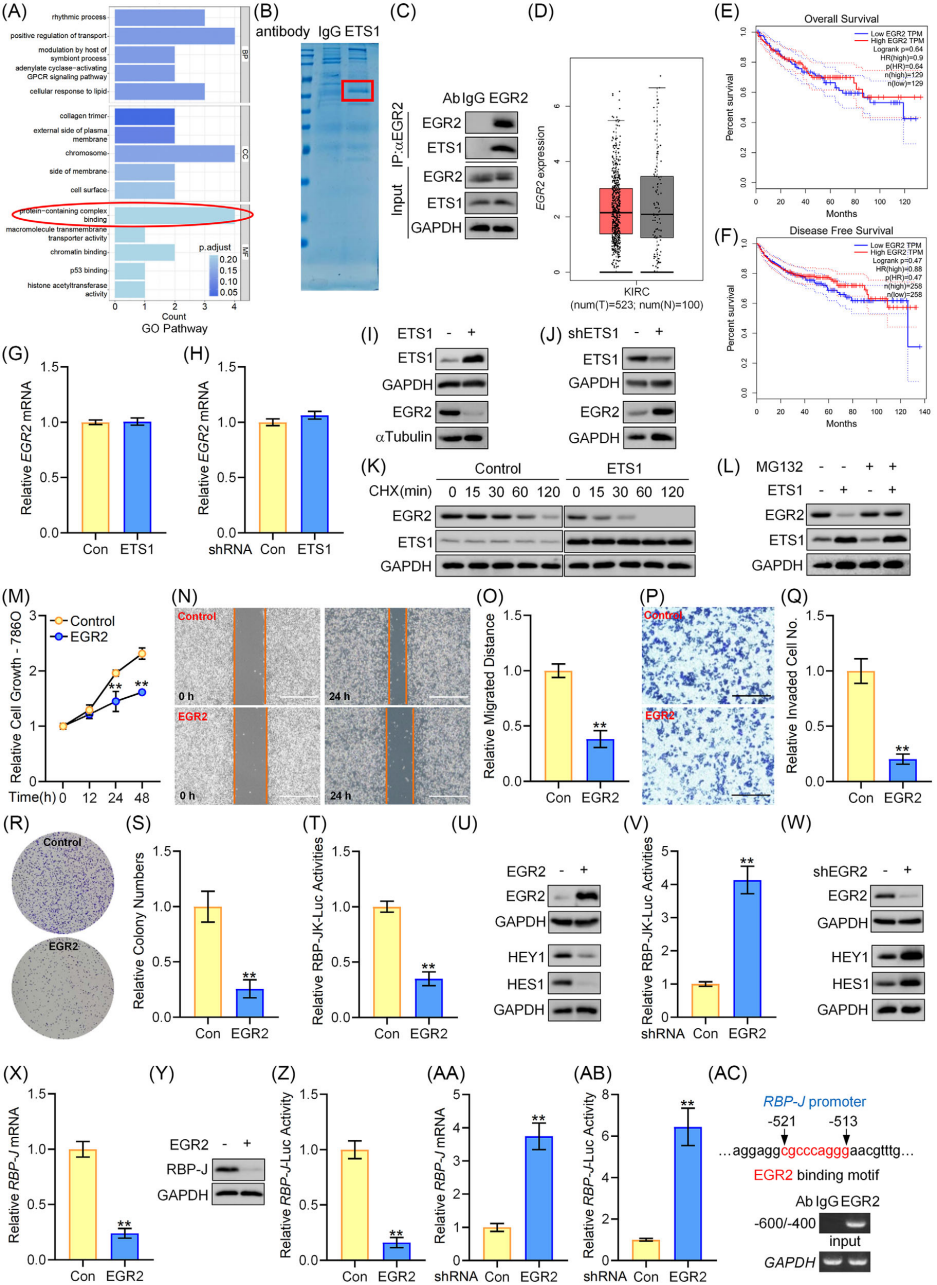
Erythroblast transformation‐specific 1 (ETS1) binds to early growth response 2 (EGR2) and destabilises EGR2. (A) GO analysis was conducted to compare the gene expression from ETS1‐overexpression group and the control. (B) Results of protein mass‐spectrum using ETS1‐immunoprecipitated sample. (C) Co‐immunoprecipitation of endogenous EGR2 and ETS1 in 786O cells. Immunoprecipitation (IP): EGR2; Western blotting (WB): ETS1. Immunoglobulin G (IgG) was used as a negative control for EGR2 antibody. (D) Expression of *EGR2* in KIRC tissues (T) and control groups (N) was revealed by GEPIA database. (E) Kaplan‒Meier analysis of overall survival for KIRC patients with altered expression of *EGR2*. (F) Kaplan‒Meier analysis of disease free survival for KIRC patients with altered expression of *EGR2*. (G) The mRNA levels of *EGR2* in 786O cells transfected with an ETS1‐expressing vector (ETS1) or a control empty vector (Con) for 24 h. (H) The mRNA levels of *EGR2* in 786O cells transfected with an ETS1 shRNA‐expressing vector (shRNA ETS1) or a control empty vector (shRNA Con) for 48 h. (I) The protein levels of ETS1 and EGR2 in 786O cells transfected with an ETS1‐expressing vector (ETS1, +) or a control empty vector (ETS1, ‒) for 24 h. (J) The protein levels of ETS1 and EGR2 in 786O cells transfected with an ETS1 shRNA‐expressing vector (shETS1, +) or a control empty vector (shETS1, ‒) for 48 h. (K) The protein levels of ETS1 and EGR2 in 786O cells transfected with an ETS1‐expressing vector (ETS1) or a control empty vector (control) for 24 h and treated for different time periods with cycloheximide (CHX). (L) The protein levels of ETS1 and EGR2 in 786O cells transfected with an ETS1‐expressing vector (ETS1, +) or a control empty vector (ETS1, ‒) for 24 h and treated with or without MG132. (M) Cell‐counting kit‐8 (CCK‐8) assays of 786O cells transfected with an EGR2‐expressing vector (EGR2) or a control empty vector (control) and cultured for the indicated time periods. (N) Wound healing assays of 786O cells transfected with an EGR2‐expressing vector (EGR2) or a control empty vector (control) at 24 h. Bar, 100 µm. (O) Statistical analysis of migrated distance in (N). (P) Matrigel invasion assays of 786O cells transfected with an EGR2‐expressing vector (EGR2) or a control empty vector (control) at 24 h. Bar, 100 µm. (Q) Statistical analysis of invaded cell number in (P). (R) Colony formation assays of 786O cells infected with lentiviruses expressing EGR2 or with control lentiviruses (control). (S) Quantitative analysis of relative colony numbers in (R). (T) RBP‐JK luciferase (RBP‐JK‐Luc) activities in 786O cells transfected with EGR2 or an empty vector (Con). (U) The protein levels of EGR2, HES1 and HEY1 in 786O cells transfected with an EGR2‐expressing vector (EGR2, +) or a control empty vector (EGR2, ‒) for 24 h. (V) RBP‐JK luciferase (RBP‐JK‐Luc) activities in 786O cells transfected with EGR2 shRNA or control shRNA. (W) The protein levels of EGR2, HES1 and HEY1 in 786O cells transfected with an EGR2 shRNA‐expressing vector (shEGR2, +) or a control empty vector (shEGR2, ‒) for 48 h. (X) The mRNA levels of *RBP‐J* in 786O cells transfected with an EGR2‐expressing vector (EGR2) or a control empty vector (Con) for 24 h. (Y) The protein levels of RBP‐J in 786O cells transfected with an EGR2‐expressing vector (EGR2) or a control empty vector (Con) for 24 h. (Z) *RBP‐J*‐promoter luciferase (*RBP‐J*‐Luc) activities in 786O cells transfected with EGR2 or an empty vector (Con). (AA) The mRNA levels of *RBP‐J* in 786O cells transfected with an EGR2 shRNA‐expressing vector (shRNA EGR2) or a control empty vector (shRNA Con) for 48 h. (AB) *RBP‐J*‐promoter luciferase (*RBP‐J*‐Luc) activities in 786O cells transfected with EGR2 shRNA‐expressing vector (shRNA EGR2) or a control empty vector (shRNA Con). (AC) Chromatin immunoprecipitation‐PCR (ChIP‐PCR) assays in 786O cells for detection of the interaction between EGR2 and ‒600/‒400 region of *RBP‐J* promoter. A group with IgG was set a negative control. *GAPDH* is amplified as a loading control. Ab, antibody. ^**^ *p* < .01; error bar, SD.

Results of further KEGG analyses indicated that ETS1 is also involved in protein binding (Figure 3A). ETS1‐associated proteins were examined using protein MS using ETS1 antibody‒immunoprecipitated protein mixture samples, and results indicated that EGR2 is a potential interacting protein (Figure 3B). The binding of EGR2 to ETS1 was validated using co‐IP assays, which showed that endogenous ETS1 was associated with EGR2 in RCC cells and visa verse (Figures 3C and S3A). However, data from a GST pull‐down assay unexpectedly indicated indirect binding between ETS1 and EGR2 (Figure S3B). Intriguingly, in contrast to that of *ETS1*, there was no difference in mRNA expression of *EGR2* between KIRC tissues (T) and normal tissues (N) (Figure 3D), as revealed by the GEPIA database; interestingly, downregulated EGR2 expression was correlated with both a lower overall survival (OS) and lower DFS in patients with KIRC (Figure 3E,F); however, the effect did not reach statistical significance. These results indicate an anti‐tumour effect of EGR2 in KIRC. Although neither overexpression nor knockdown of ETS1 affected *EGR2* mRNA expression (Figure 3G,H), the ectopic expression of ETS1 effectively suppressed EGR2 protein expression, and silencing of ETS1 increased EGR2 protein levels (Figure 3I,J), indicating that ETS1 binds to EGR2 and modulates EGR2 at least at the protein level. Indeed, data from the CHX‐chase assay showed that ETS1 overexpression significantly shortened the half‐life of EGR2 (Figures 3K and S3C,D), and the ETS1‐directed decrease in EGR2 protein expression was almost completely reversed by the addition of the proteasome inhibitor MG132^31^ (Figure 3L). Consistently, ETS1 overexpression potentiated EGR2 ubiquitination (Figure S3E), demonstrating that ETS1 disrupted EGR2 protein stability by inducing its degradation. As expected, EGR2 overexpression significantly reduced RCC cell proliferation at 24 and 48 h (Figure 3M), inhibited the migratory and invasive potential of RCC cells (Figure 3N‒Q), and decreased the colony number (Figure 3R,S), which is consistent with a previous report showing that EGR2 inhibits tumour cell growth by inducing apoptosis.^32^ Consistently, EGR2 overexpression not only significantly suppressed RBP‐JK‐Luc reporter activity, but also downregulated the protein expression of targets HES1 and HEY1 (Figure 3T,U), whereas EGR2 knockdown promoted RBP‐JK‐Luc reporter activity and potentiated HES1 and HEY1 expression (Figure 3V,W), proving that EGR2 negatively regulates the NOTCH signalling pathway. Consistently, Western blotting results revealed that EGR2 protein expression was significantly decreased in tumour samples compared to that in the normal control group (Figure S3F).

RBP‐J is a key transcription factor regulating NOTCH activity,^33^ and RBP‐J overexpression significantly increases the mRNA expression of *HES1* and *HEY1* (Figure S3G‒I). EGR2 overexpression not only suppressed the mRNA and protein expression of RBP‐J but also inhibited *RBP‐J*‐promoter luciferase activity (Figure 3X‒Z), whereas EGR2 knockdown significantly increased the mRNA expression of *RBP‐J* and induced *RBP‐J*‐promoter luciferase activity (Figure 3AA, AB). Intriguingly, a potential binding motif for EGR2 was detected within the promoter region of *RBP‐J* (‐521/‐513), and the direct association between EGR2 and *RBP‐J* promoter was confirmed using a ChIP assay (Figure 3AC), indicating that EGR2 reduces NOTCH signalling activity by suppressing *RBP‐J* transactivation.

### 3.4 EGR2 restrains RCC cell biological malignancies by deactivating the NOTCH signalling

**FIGURE 4:**
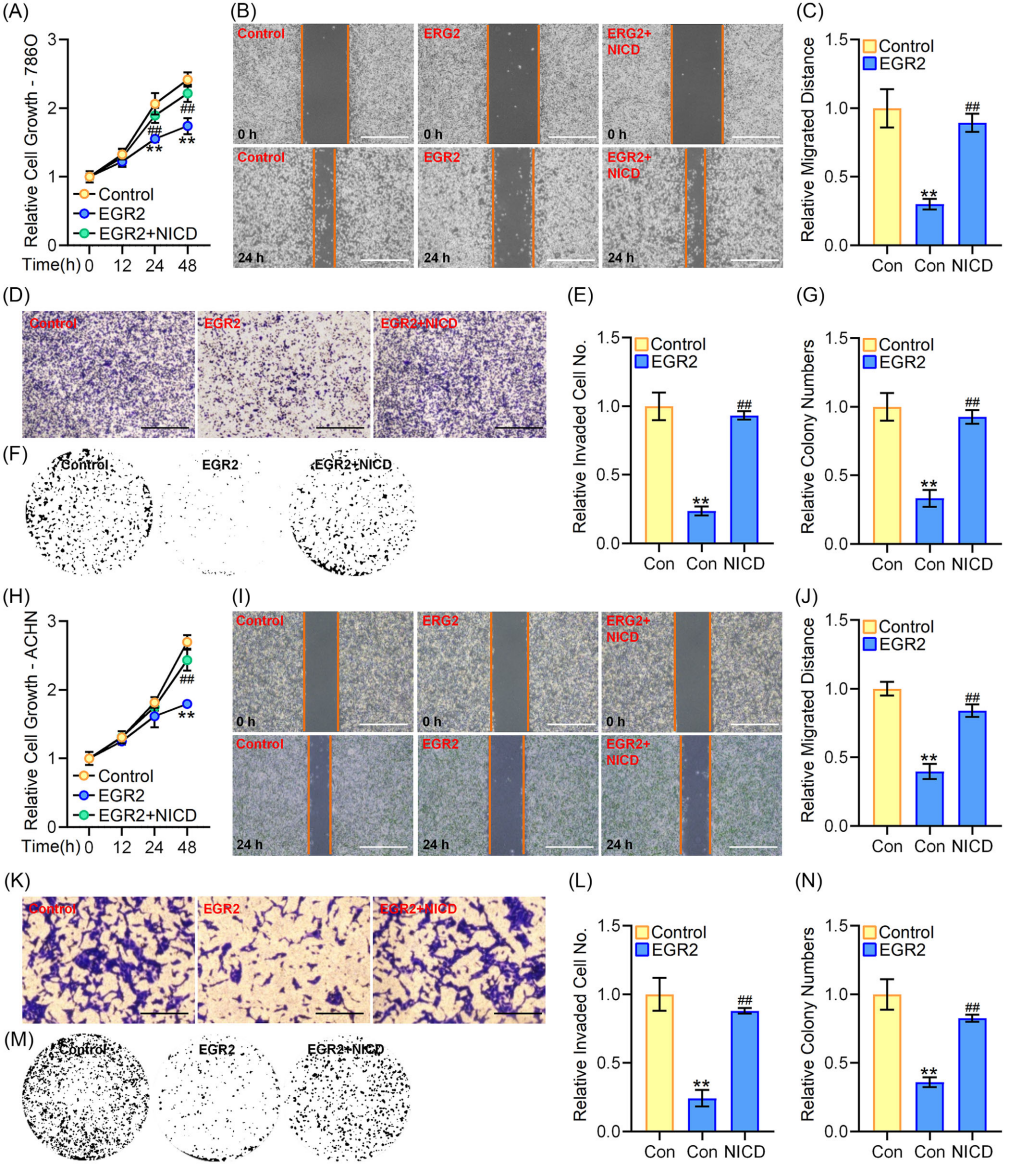
Early growth response 2 (EGR2) restrains renal cell carcinoma (RCC) cell biological malignancies by deactivating the NOTCH signalling. (A) Cell‐counting kit‐8 (CCK‐8) assays of 786O cells transfected with an EGR2‐expressing vector (EGR2) in combination with a NOTCH1 intracellular domain (NICD)‐expression vector (NICD) or a control empty vector (control) and cultured for the indicated time periods. (B) Wound healing assays of 786O cells transfected with an EGR2‐expressing vector (EGR2) in combination with a NICD‐expression vector (NICD) or a control empty vector (control) at 24 h. Bar, 100 µm. (C) Statistical analysis of migrated distance in (B). (D) Matrigel invasion assays of 786O cells transfected with an EGR2‐expressing vector (EGR2) in combination with a NICD‐expression vector (NICD) or a control empty vector (control) at 24 h. Bar, 100 µm. (E) Statistical analysis of invaded cell number in (D). (F) Colony formation assays of 786O cells infected with lentiviruses expressing EGR2 in combination with lentiviruses expressing NICD or with control lentiviruses (control). (G) Quantitative analysis of relative colony numbers in (F). (H) CCK‐8 assays of ACHN cells transfected with an EGR2‐expressing vector (EGR2) in combination with a NICD‐expression vector (NICD) or a control empty vector (control) and cultured for the indicated time periods. (I) Wound healing assays of ACHN cells transfected with an EGR2‐expressing vector (EGR2) in combination with a NICD‐expression vector (NICD) or a control empty vector (control) at 24 h. Bar, 100 µm. (J) Statistical analysis of migrated distance in (I). (K) Matrigel invasion assays of ACHN cells transfected with an EGR2‐expressing vector (EGR2) in combination with a NICD‐expression vector (NICD) or a control empty vector (Control) at 24 h. Bar, 100 µm. (L) Statistical analysis of invaded cell number in (K). (M) Colony formation assays of ACHN cells infected with lentiviruses expressing EGR2 in combination with lentiviruses expressing NICD or with control lentiviruses (control). (N) Quantitative analysis of relative colony numbers in (M). ^**^ *p* < .01; ^##^ *p* < .01; error bar, SD.

To confirm whether EGR2‐derived suppressive effects on RCC cells is dependent on deactivation of the NOTCH signalling, we next overexpressed EGR2 and simultaneously transfected with NICD to activate downstream of NOTCH signalling. The data showed that ectopic expression of NICD effectively reversed the inhibitory effects of EGR2‐overexpression on RCC cell proliferation, migration, invasion and colony formation, with the enhanced cell proliferation (Figure 4A), elevated migration and invasion as well as the increased colony number (Figure 4B‒G). Consistently, the negative effect of EGR2 on RCC cell oncogenic malignancies through deactivating NOTCH signalling could be additionally verified in another RCC cell line where EGR2 expression is relatively low (Figure S4A). Experimental data illustrated that forced NICD expression could markedly reverse the inhibitory phenotypes induced by EGR2 in RCC cells. Specifically, NICD overexpression counteracted EGR2‐mediated suppression of cell multiplication, migratory capacity, invasive potential and clonogenic growth, thereby accelerating cellular proliferative activity (Figure 4H), potentiating migration and invasion and upregulating colony number (Figure 4I‒N). Thus, these data reinforce the notion that EGR2 suppresses RCC progression possibly through deactivating the NOTCH signalling pathway.

### 3.5 Deactivation of NOTCH restores ETS1‐triggered tumour‐stimulatory effects

**FIGURE 5:**
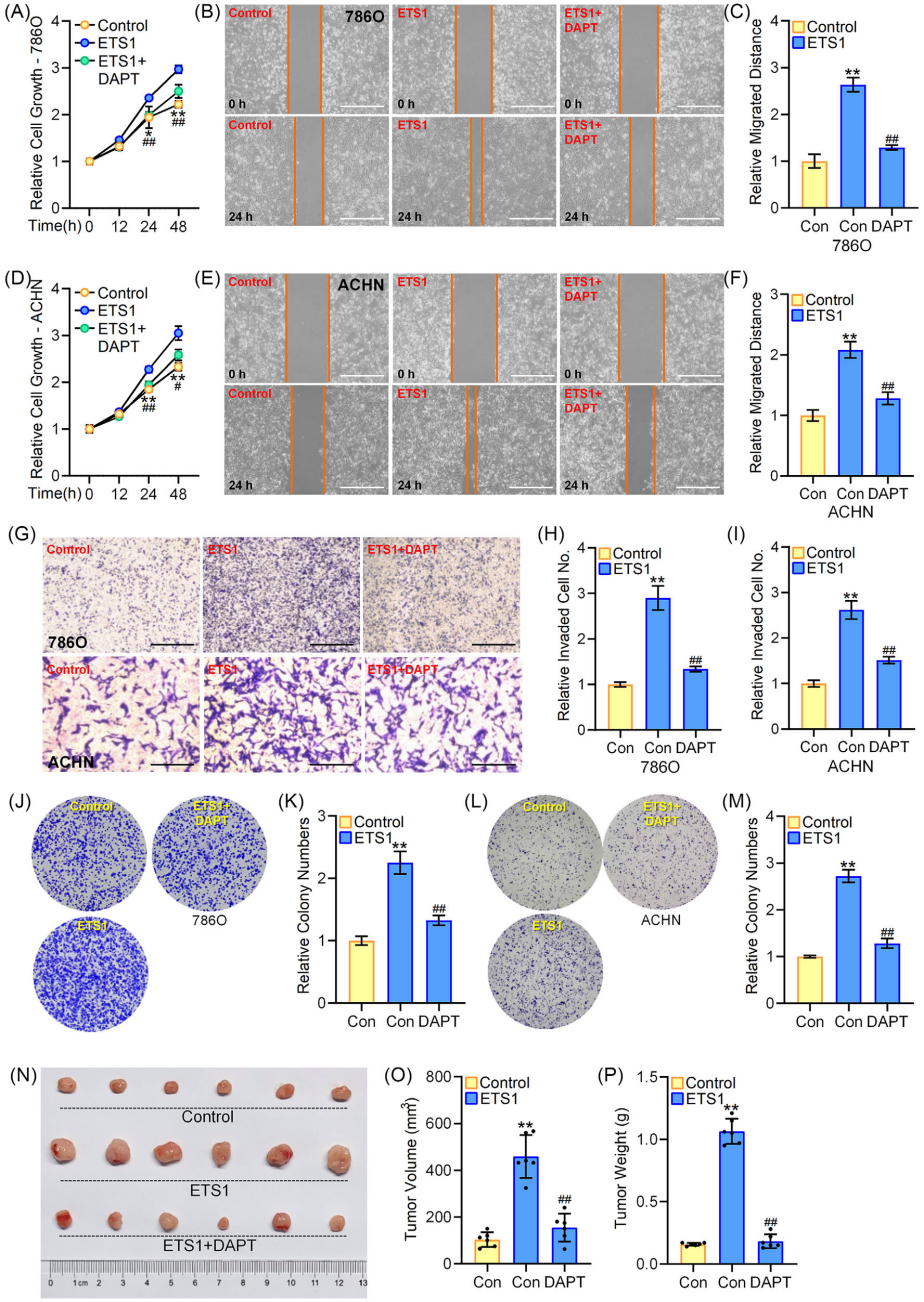
Erythroblast transformation‐specific 1 (ETS1) stimulates renal cell carcinoma (RCC) progression through activating the NOTCH signalling. (A) Cell‐counting kit‐8 (CCK‐8) assays of 786O cells transfected with an ETS1‐expressing vector (ETS1) and cultured with or without DAPT for the indicated time periods. (B) Wound healing assays of 786O cells transfected with an ETS1‐expressing vector (ETS1) and cultured with or without DAPT for 24 h. Bar, 100 µm. (C) Statistical analysis of migrated distance in (B). (D) CCK‐8 assays of ACHN cells transfected with an ETS1‐expressing vector (ETS1) and cultured with or without DAPT for the indicated time periods. (E) Wound healing assays of ACHN cells transfected with an ETS1‐expressing vector (ETS1) and cultured with or without DAPT for 24 h. Bar, 100 µm. (F) Statistical analysis of migrated distance in (E). (G) Matrigel invasion assays of 786O cells (upper) and ACHN cells (lower) transfected with an ETS1‐expressing vector (ETS1) and cultured with or without DAPT for 24 h. Bar, 100 µm. (H) Statistical analysis of invaded 786O cell number in (G, upper). (I) Statistical analysis of invaded ACHN cell number in (G, lower). (J) Colony formation assays of 786O cells infected with lentiviruses expressing ETS1 in combination with or without DAPT treatment. (K) Quantitative analysis of relative colony numbers in (J). (L) Colony formation assays of ACHN cells infected with lentiviruses expressing ETS1 in combination with or without DAPT treatment. (M) Quantitative analysis of relative colony numbers in (L). (N) Xenografts derived from 786O cells stably expressing ETS1 in combination with or without DAPT administration. (O) The volume of xenografts from different groups in (N). (P) The weight of xenografts from different groups in (N). ^*^ *p* < .05; ^#^ *p* < .05; ^**^ *p* < .01; ^##^ *p* < .01; error bar, SD.

To test whether ETS1 stimulates RCC progression through activating NOTCH, we next observed the cell malignant behaviours in the presence of ETS1 and co‐treatment with DAPT, an antagonist against NOTCH signalling.^34^ While overexpression of ETS1 significantly increased proliferation, migration, invasion and colony numbers in both 786O and ACHN cells (Figure 5A‒M), DAPT treatment effectively alleviated the positive role of ETS1 on cell biological functions (Figure 5A‒M). Consistently, administration of DAPT significantly reduced the volume and weight of 786O cell‐derived xenografts in nude mice, which was induced by ETS1‐overexpression (Figure 5N‒P). Therefore, ETS1 promotes RCC progression through activating NOTCH signalling.

### 3.6 ETS1‒EGR2‐mediated NOTCH activation promotes RCC progression in vivo

**FIGURE 6:**
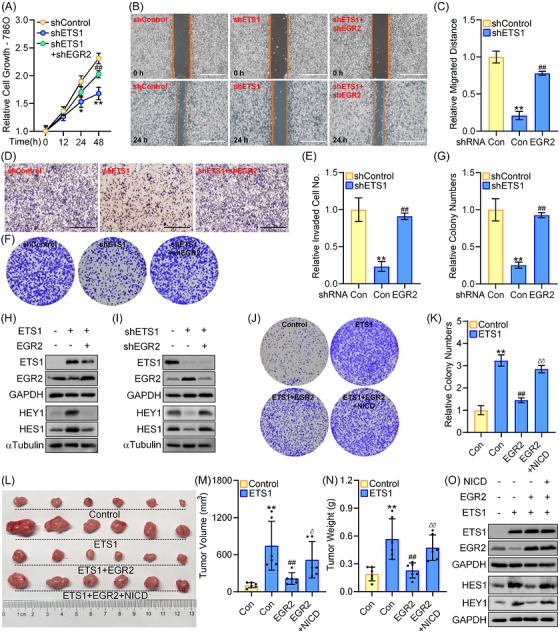
Erythroblast transformation‐specific 1 (ETS1)‒early growth response 2 (EGR2)‐mediated NOTCH activation promotes renal cell carcinoma (RCC) progression in vivo. (A) Cell‐counting kit‐8 (CCK‐8) assays of 786O cells transfected with an ETS1 shRNA‐expressing vector (shETS1) in combination with an EGR2 shRNA‐expression vector (shEGR2) or a control empty vector (shControl) and cultured for the indicated time periods. (B) Wound healing assays of 786O cells transfected with an ETS1 shRNA‐expressing vector (shETS1) in combination with an EGR2 shRNA‐expression vector (shEGR2) or a control empty vector (shControl) at 24 h. Bar, 100 µm. (C) Statistical analysis of migrated distance in (B). (D) Matrigel invasion assays of 786O cells transfected with an ETS1 shRNA‐expressing vector (shETS1) in combination with an EGR2 shRNA‐expression vector (shEGR2) or a control empty vector (shControl) at 24 h. Bar, 100 µm. (E) Statistical analysis of invaded cell number in (D). (F) Colony formation assays of 786O cells infected with lentiviruses expressing ETS1 shRNA (shETS1) in combination with lentiviruses expressing EGR2 shRNA (shEGR2) or with control lentiviruses (control). (G) Quantitative analysis of relative colony numbers in (F). (H) The protein levels of ETS1, EGR2, HES1 and HEY1 in 786O cells transfected with an ETS1‐expressing vector (ETS1) in combination with an EGR2‐expression vector (EGR2) or a control empty vector for 24 h. (I) The protein levels of ETS1, EGR2, HES1 and HEY1 in 786O cells transfected with an ETS1 shRNA‐expressing vector (shETS1) in combination with an EGR2 shRNA‐expression vector (shEGR2) or a control empty vector for 48 h. (J) Colony formation assays of 786O cells infected with lentiviruses expressing ETS1 in combination with lentiviruses expressing EGR2 and NOTCH1 intracellular domain (NICD) or with control lentiviruses (control). (K) Quantitative analysis of relative colony numbers in (J). (L) Xenografts derived from 786O cells stably expressing ETS1 in combination with lentiviruses expressing EGR2 and NICD or with control viruses. (M) The volume of xenografts from different groups in (L). (N) The weight of xenografts from different groups in (L). (O) The protein levels of ETS1, EGR2, HES1 and HEY1 of xenografts from different groups in (L). ^∂^ *p* < .05; ^**^ *p* < .01; ^##^ *p* < .01; ^∂∂^ *p* < .01; error bar, SD.

Given the regulation of EGR2 by ETS1 (Figure 3), we aimed to clarify whether ETS1 potentiates RCC progression by suppressing EGR2. Silencing endogenous ETS1 expression restrained the proliferative, migratory and invasive potential of renal carcinoma cells and diminished their clonogenic capacity (Figure 6A‒G). This effect was significantly attenuated by silencing EGR2, resulting in increased proliferation, improved migratory and invasive capacities, and upregulated colony number (Figure 6A‒G). Consistently, EGR2 knockdown effectively restored the negative regulation of RBP‐JK‐Luc reporter activity as well as the expression of HES1 and HEY1 via ETS1 knockdown (Figures 6H,I and S5A), whereas EGR2 overexpression significantly suppressed ETS1‐induced RBP‐JK‐Luc reporter activity in both 786O and ACHN cells (Figure S5B,C). Conversely, EGR2 overexpression markedly suppressed RCC cell biological behaviour, including colony number (Figure 6J,K), which was alleviated by co‐overexpression of NICD (Figure 6J,K), highlighting the role of the ETS1‒EGR2‒NOTCH axis in RCC cell function. Consistently, compared to the control group, ETS1 overexpression by lentiviral infection promoted tumour growth, including both tumour volume and weight, as well as the expression of HES1 and HEY1 in tumour tissues (Figure 6L‒O), which was almost reversed by EGR2 overexpression (Figure 6L‒O). However, co‐overexpression of NICD revealed significant alleviative effects on the EGR2‐derived suppression on tumour growth and expression of HES1 and HEY1 in tumours (Figure 6L‒O). In summary, our data demonstrate that the ETS1‒EGR2 axis, through the NOTCH pathway, acts as an important and positive mediator in RCC progression in vivo.

## 4 DISCUSSION

**FIGURE 7:**
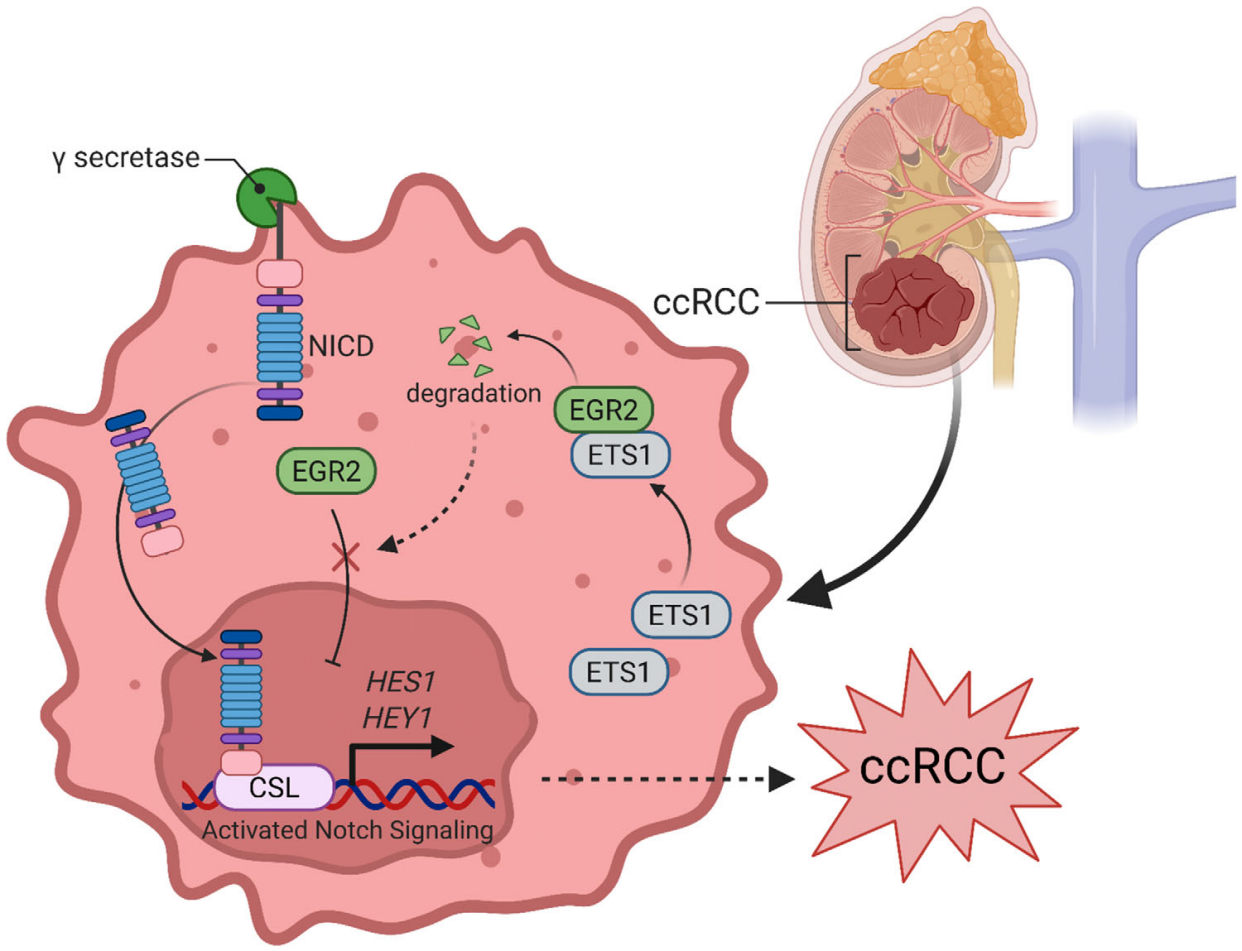
Working model summarising the mechanisms underlying erythroblast transformation‐specific 1 (ETS1)‒ early growth response 2 (EGR2)‒NOTCH axis in renal cell carcinoma (RCC) progression.

Using the canonical VHL‐mutated ccRCC cell line 786O and papillary RCC cell line ACHN, we found that ETS1 expression is significantly elevated in ccRCC tissues, and that ETS1 increases the biological activity of RCC cells, including proliferation, invasion and colony formation in vitro and promotes RCC progression in a nude mouse xenograft model in vivo. Mechanistically, ETS1 forms a complex with EGR2 and destabilises EGR2 by inducing its degradation, and the decreased EGR2 levels lead to the activation of the NOTCH signalling pathway via transactivation of *RBP‐J*, resulting in RCC development (Figure 7). Therefore, targeting the ETS1‒EGR2‒NOTCH signalling axis may potentially be a promising therapeutic strategy for RCC.

As a member of the ETS family, ETS1 has been shown to be closely associated with the development of various cancers, such as breast cancer,^35^ non‐small cell lung cancer,^36^ and leukaemia and lymphoma,^37^ with the reported diverse function of ETS1 in cancer cell function. A previous report showed that ETS1 directly regulates drug transporters such as MDR1 in gastric SGC7901 cells,^38^ and research conducted by Dhaha and colleagues uncovered that ETS1 serves as a transcriptional activator for genes belonging to E2F, MYC as well as G2/M signalling cascades.^35^ This regulatory function ultimately boosts DNA replication within triple‐negative breast cancer cells. More recently, a study reported that ETS1 facilitates epithelial‐to‐mesenchymal transition through DNM1L (DRP1)‐mediated mitochondrial fragmentation in ovarian cancer.^39^ In this study, using sequencing and various experimental methods, we further revealed that ETS1 activates NOTCH signalling, leading to RCC progression, which complements the role of ETS1 in predicting cancer and for targeted therapy. Nevertheless, the clinical sample size used in this study was small, making the current clinical results preliminary exploratory findings; therefore, a larger sample size is needed for further validation.

Given the correlation of ETS1 with different cancers, investigations have been conducted to determine the possible mechanisms underlying elevated ETS1 expression in cancer. Kim and colleagues characterised a core regulatory element (CRE) within the ETS1 promoter spanning positions −540 to −80 bp relative to the transcription start site. This region harbours binding motifs for NFAT and NF‐κB family transcription factors. Further functional validation demonstrated that NFATc2 and NFKB1/RELA jointly activate ETS1 transcription, and this ETS1 upregulation drives invasive phenotypes in breast cancer cells.^40^ In addition, the ETS1 protein is modified by sumoylation and ubiquitination, wherein sumoylation of ETS1 does not affect its stability but leads to reduced transactivation.^41^ Despite these data, the underlying mechanisms of *ETS1* expression remain ambiguous, and the aberrant upregulation of ETS1 expression in RCC requires further exploration.

EGR2 was initially characterised as a serum‐inducible immediate early gene encoding a polypeptide with three conserved Cys2‒His2 zinc finger motifs.^32^ Accumulating evidence indicates that EGR2 functions as a suppressor of tumour progression. One prior study illustrated that EGR2 impairs the survival, migration and invasion of HepG2 cells and triggers cell apoptosis, an effect attributed to reduced phosphorylated AKT and ERK protein abundance.^42^ Consistent with these findings, another research demonstrated that EGR2 exerts suppressive effects on papillary thyroid carcinoma cell proliferation. Mechanistically, EGR2 directly interacts with the promoter sequences of *PTEN* and *BAX* to repress their transcription, thereby restraining tumour cell proliferation.^43^ More recently, Symonds et al. found that, deficiency of *Egr2* in T cells resulted in enhanced tumour growth and fewer tumour infiltrating T cells (TILs) in mouse models, indicating an important role of EGR2 in maintaining anti‐tumour responses of exhausted tumour infiltrating T cells.^44^ Consistently, in this study, we report that EGR2 restrains RCC cell proliferation, migration, invasion and colony formation through deactivation of NOTCH signalling pathway. Moreover, we have clarified the possible mechanisms underlying how EGR2 regulates NOTCH signalling activity in this study. EGR2 directly binds to *RBP‐J* promoter, which encodes RBP‐J protein, a key transcriptional factor regulating NOTCH activity, and suppresses its transactivation. Nevertheless, whether the molecular mechanisms exist in other different cancers remain yet unclear, which warrants further investigation.

Another limitation of the current study should be also noted. Our in vivo tumour validation was limited to subcutaneous 786O cell line xenografts, which cannot fully recapitulate the intra‐tumoural heterogeneity, complex stromal microenvironment and metastatic progression characteristics of clinical ccRCC. Patient‐derived xenograft (PDX) models and orthotopic/lung metastatic models are superior preclinical platforms to bridge basic mechanistic discoveries and clinical translation. Due to the long experimental cycle required for PDX establishment and metastatic model characterisation, these animal models could not be fully constructed and tested within the current study. Therefore, subsequent independent studies verifying the regulatory axis in PDX and metastatic in vivo systems are worthy of investigation to obtain more direct translational evidence.

In conclusion, the current study substantiates the critical role of the ETS1‒EGR2 axis in NOTCH signalling pathway transduction in RCC cells. The ETS1‒EGR2‒NOTCH pathway may provide therapeutic targets for the treatment of RCC in future clinical practice.

## AUTHOR CONTRIBUTIONS

Weifeng Ye, Sishun Gan and Qiang Xu performed all the experiments and prepared the figures. Xiaoyang Shu and Panpan Dong analysed the data and revised the manuscript. Lin Li and Chao Tang designed the study and wrote the manuscript text. All the authors reviewed the manuscript.

## CONFLICT OF INTEREST STATEMENT

The authors declare they have no conflicts of interest.

## ETHICS STATEMENT

The Ethics Committee of Third Affiliated Hospital of Naval Medical University approved the protocols of the study in accordance with the Declaration of Helsinki. All participants provided written informed consent for the use of ccRCC tissues and adjacent non‐tumour tissues.

## CONSENT FOR PUBLICATION

The author declares that all work described here has not been published before and that its publication has been approved by all co‐authors.

## AI USE

The authors declare no AI use in any part of the manuscript or figure preparation.

## Supporting information




**Figure S1**. (A) GSEA data showing the enriched biological process in erythroblast transformation‐specific 1 (ETS1)‐high tumour samples. (B) GSEA data showing the enriched biological process in ETS1‐low tumour samples. (C) The mRNA levels of *ETS1* in 786O cells transfected with an ETS1 shRNA‐expressing vector (shRNA ETS1) or a control empty vector (shRNA Con) for 48 h. (D) The protein levels of ETS1 in 786O cells transfected with an ETS1 shRNA‐expressing vector (shRNA ETS1) or a control empty vector (shRNA Con) for 48 h. (E) The protein levels of ETS1 in *ETS1*‐knockout 786O cells (*ETS1* ^KO^). (F) Cell‐counting kit‐8 (CCK‐8) assays of *ETS1*‐knockout 786O cells (*ETS1* ^KO^) cultured for the indicated time periods. (G) Wound healing assays of *ETS1*‐knockout 786O cells (*ETS1* ^KO^). Bar, 100 µm. (H) Statistical analysis of migrated distance in (E). (I) Matrigel invasion assays of *ETS1*‐knockout 786O cells (*ETS1* ^KO^). Bar, 100 µm. (J) Statistical analysis of invaded 786O cell number in (G). (K) Colony formation assays of *ETS1*‐knockout 786O cells (*ETS1* ^KO^). (L) Quantitative analysis of relative colony numbers in (I). ^**^ *p* < .01; error bar, SD.

**Figure S2**. (A) Number of genes whose expression was altered by ectopic expression of erythroblast transformation‐specific 1 (ETS1) is summarised in the table. (B) GSEA data showing the enriched signalling in ETS1‐high tumour samples. (C) GSEA data showing the enriched signalling in ETS1‐low tumour samples. (D) RBP‐JK luciferase (RBP‐JK‐Luc) activities in 786O cells transfected with ETS1 or an empty vector (Con). (E) RBP‐JK luciferase (RBP‐JK‐Luc) activities in 786O cells transfected with ETS1 shRNA or control shRNA. ^**^ *p* < .01; error bar, SD.

**Figure S3**. (A) Co‐immunoprecipitation of endogenous early growth response 2 (EGR2) and erythroblast transformation‐specific 1 (ETS1) in 786O cells. Immunoprecipitation (IP): ETS1; Western blotting (WB): EGR2. Immunoglobulin G (IgG) was used as a negative control for ETS1 antibody. (B) GST pull‐down assays for recombinant GST‒ETS1 protein and Flag‐EGR2‐expressing cell lysates. (C) The protein levels of EGR2 in 786O cells transfected with an ETS1‐expressing vector (ETS1) or a control empty vector (control) for 24 h and treated for different time periods with cycloheximide (CHX). Results from repeated experiments were shown. (D) Quantitative analysis of relative EGR2 protein expression in (C). (E) Co‐immunoprecipitation (Co‐IP) of endogenous EGR2 and ubiquitin (Ub) in 786O cells transfected with ETS1 (ETS1, +) or control (ETS1, ‒). IP: EGR2; WB: Ub. (F) The protein expression of EGR2 in cancer tissues (tumour) and adjacent normal epithelial tissues (normal) from patients with clear‐cell renal cell carcinoma (ccRCC). (G) Protein levels of Flag (RBP‐J) in 786O cells transfected with Flag‐RBP‐J or a control empty vector for 24 h. (H) The mRNA levels of *HES1* in 786O cells transfected with Flag‐RBP‐J or a control empty vector for 24 h. (I) The mRNA levels of *HEY1* in 786O cells transfected with Flag‐RBP‐J or a control empty vector for 24 h.
**p < .01; error bar, SD.

**Figure S4**. (A) Expression analysis of *EGR2* in different renal cell carcinoma (RCC) cell lines from the Cancer Cell Line Encyclopaedia (CCLE) portal. The graph presents a bar chart of gene expression distribution across different RCC cell lines. The horizontal axis represents the expression level of the gene *EGR2*, while the vertical axis lists the different cell lines. Both the height and colour of the bars indicate the magnitude of *EGR2* gene expression, with the median serving as the cutoff point.

**Figure S5**. (A) RBP‐JK luciferase (RBP‐JK‐Luc) activities in 786O cells transfected with an erythroblast transformation‐specific 1 (ETS1) shRNA‐expressing vector (shETS1) in combination with an early growth response 2 (EGR2) shRNA‐expression vector (shEGR2) or a control empty vector (shControl) at 24 h. (B) RBP‐JK luciferase (RBP‐JK‐Luc) activities in 786O cells transfected with ETS1 in combination with EGR2 or an empty vector (Con). (C) RBP‐JK luciferase (RBP‐JK‐Luc) activities in ACHN cells transfected with ETS1 in combination with EGR2 or an empty vector (Con). ^**^ *p* < .01; ^##^ *p* < .01; error bar, SD.

## Data Availability

All data generated or analysed during this study are included in this published article.
